# Inpatient and outpatient costs in patients with coronary artery disease and mental disorders: a systematic review

**DOI:** 10.1186/s13030-015-0039-z

**Published:** 2015-04-17

**Authors:** Harald Baumeister, Anne Haschke, Marie Munzinger, Nico Hutter, Phillip J Tully

**Affiliations:** Department of Rehabilitation Psychology and Psychotherapy, Institute of Psychology, University of Freiburg, Engelbergerstr 41, D-79085 Freiburg, Germany; Medical Psychology and Medical Sociology, Medical Faculty, University of Freiburg, Freiburg, Germany; Freemasons Foundation Centre for Men’s Health, Discipline of Medicine, School of Medicine, The University of Adelaide, Adelaide, Australia

**Keywords:** Coronary artery disease, Mental disorder, Depression, Comorbidity, Health care costs, Systematic review

## Abstract

**Background:**

To systematically review in- and outpatient costs in patients with coronary artery disease (CAD) and comorbid mental disorders.

**Methods:**

A comprehensive database search was conducted for studies investigating persons with CAD and comorbid mental disorders (Medline, EMBASE, PsycINFO, Psyndex, EconLit, IBSS). All studies were included which allowed a comparison of in- and outpatient health care costs (assessed either monetarily or in terms of health care utilization) of CAD patients with comorbid mental disorders (mood, anxiety, alcohol, eating, somatoform and personality disorders) and those without. Random effects meta-analyses were conducted and results reported using forest plots.

**Results:**

The literature search resulted in 7,275 potentially relevant studies, of which 52 met inclusion criteria. *Hospital readmission rates* were increased in CAD patients with any mental disorder (pooled standardized mean difference (SMD) = 0.34 [0.17;0.51]). Results for depression, anxiety and posttraumatic stress disorder pointed in the same direction with heterogeneous SMDs on a primary study level ranging from −0.44 to 1.26. Length of hospital stay was not increased in anxiety and any mental disorder, while studies on depression reported heterogeneous SMDs ranging from −0.08 to 0.82. Most studies reported increased overall and outpatient costs for patients with comorbid mental disorders. Results for invasive procedures were non-significant respectively inconclusive.

**Conclusions:**

Comorbid mental disorders in CAD patients are associated with an increased healthcare utilization in terms of higher hospital readmission rates and increased overall and outpatient health care costs. From a health care point of view, it is requisite to improve the diagnosis and treatment of comorbid mental disorders in patients with CAD to minimize incremental costs.

## Introduction

Comorbid mental disorders are common in CAD patients [1-3]. A significant increase of in- and outpatient costs in patients with other physical diseases such as diabetes, asthma and back pain has been documented [4-6]. However, while CAD has been associated with increased mortality [7] and diminished quality of life [8,9], data regarding health care costs in CAD patients are inconsistent. For example, depression in patients with CAD was associated with increased readmission rates in one study (d = 0.63; 95%-CI: 0.61-0.65) [10], whereas another study reported decreased readmission rates for depressed CAD patients compared to CAD patients without depression (d = −0.44; 95%-CI: −0.81- -0.07) [11]. Hence, the aim of the present study was to systematically review the association between comorbid mental disorders and in- and outpatient costs in CAD patients. The following research questions will be addressed:

1. Are in- and outpatient costs increased in CAD patients with mental disorders compared to CAD patients without mental disorders?
2. Are there differences in this association with regard to specific mental disorder subtypes?

## Methods

Data collection for this systematic review was part of a larger systematic review on quality of life and health care costs in somatically ill patients with comorbid mental disorders [4-6,9]. The present review focusses on direct costs of CAD patients with comorbid mental disorders compared to CAD patients without mental comorbidity. The reporting of this study follows the PRISMA (Preferred Reporting Items for Systematic Reviews and Meta-Analyses) statement as detailed in the PRISMA checklist provided as supplementary document.

### Inclusion criteria

Studies investigating adult patients (≥18 years) with CAD (International Classification of Disease Criteria 10^th^ Revision [ICD-10]: I20-I25) in outpatient or inpatient settings as well as community samples were included. Inclusion of primary studies was not further limited to specific clinical subgroups in order to increase the generalizability of the results of the review.

Studies were included that allowed the categorization of mental disorders or psychological burden corresponding to the following diagnostic categories: 1) mental and behavioral disorders due to use of alcohol (ICD-10: F10; DSM-IV: 303.xx, 291.xx), 2) mood disorders (ICD-10: F30-F39; DSM-IV: 292.xx, 296.xx; 300.4, 301.13, 311), 3) anxiety disorders (ICD-10: F40-F43; DSM-IV: 300.0x, 300.2x, 308.3, 309.81), 4) somatoform disorders (ICD-10: F45; DSM-IV: 300.7, 300.81), 5) eating disorders (ICD-10: F50; DSM-IV: 307.1, 307.5x), 6) disorders of adult personality and behavior (ICD-10: F60; DSM-IV: 301.x), or 7) any mental disorder (i.e. assessment of psychiatric symptoms in general). For inclusion, primary studies had to allow for a comparison regarding health care costs between a group with one of the mentioned mental disorders, and a group without mental comorbidities.

Primary studies were included if they assessed any direct health care costs either monetarily or in terms of utilization of health care resources. Direct inpatient (hospital readmissions, invasive procedures), outpatient (physician visits, emergency room visits and rehabilitation) and other health care costs such as hospital transfer were included.

### Search strategy

The database search was conducted in Medline, EMBASE, PsycINFO, Psyndex, EconLit and IBSS for articles published until 24 February 2014 using the search structure ‘coronary artery disease’ and ‘mental disorders’ and ‘health care costs/health care utilization’. The comprehensive search strategy for Medline can be requested from the first author.

**Figure 1:**
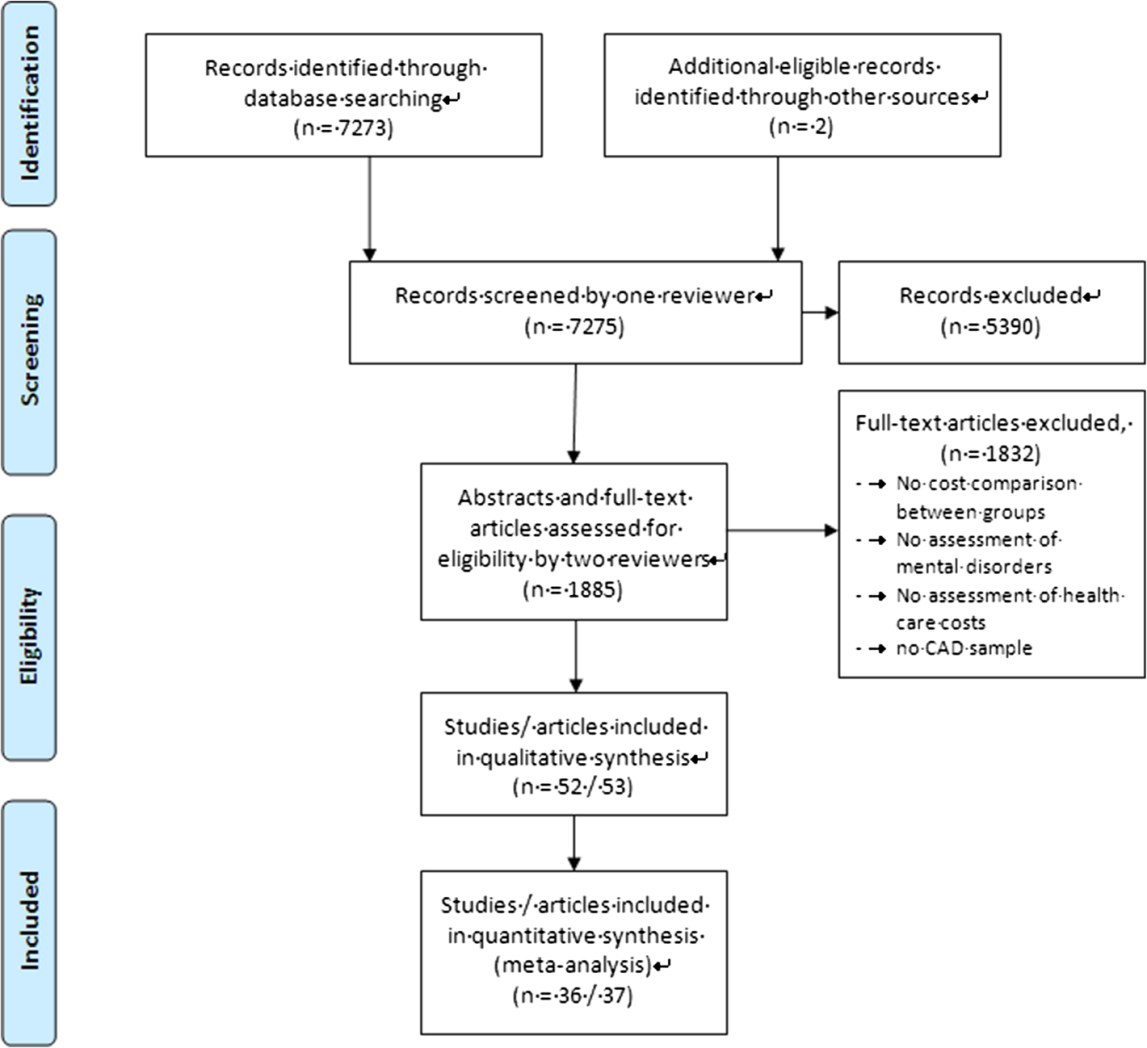
Selection process of primary studies.

In a preliminary sensitive selection process, one reviewer (AH or MM) screened titles and abstracts of English- or German-language articles relating to cost studies in CAD (N = 7,273) (Figure 1). Then, two reviewers (two out of HB, AH, MM, NH) independently selected relevant studies for inclusion by examining the remaining titles, abstracts or full papers (N = 1,883). In the case of disagreement, a third reviewer of the author team was asked to review the article, and disagreements were solved by consensus discussion. When multiple articles were published on the same study sample, the most comprehensive paper was selected as reference article. Further potentially relevant studies were retrieved by examining the reference lists of included studies and through an identification of published articles citing included studies (Web of Science Cited Reference Search). In addition, experts in the area were contacted and asked about published or unpublished studies that are relevant to the review.

### Data abstraction

Two reviewers (two out of HB, AH, MM, NH) extracted data from primary studies independently using a data extraction form. Information about participants (sample size, sex and age), type of CAD, mental disorder, assessment method of mental disorders (standardized diagnostic interview, self-report questionnaire, medical record or physician’s diagnosis), cut-off scores used to indicate mental disorders on self-report questionnaires, means and standard deviations of mental disorder scores and descriptive statistics of outcomes were extracted.

### Quantitative data analysis

The data analysis was completed using Stata Statistical Software 9.0 (StataCorp, College Station, Texas, USA) and Review Manager 5.0 (Nordic Cochrane Centre, Copenhagen, Denmark). Standardised mean differences (SMD as Hedges’ g) with 95%-CIs using the pooled standard deviation of both groups for continuous data and Odds ratios (OR) (95%-CI) for dichotomous data were computed.

In studies examining more than two groups representing different grades of severity of specific mental disorders (e.g. no depression, minor depression, and major depression), the groups of patients with psychiatric symptoms were merged and compared to patients without psychiatric symptoms (e.g. no depression vs. minor and major depression). If no measures of variability were given in study reports, p-values were used to compute effect sizes.

**Figure 2:**
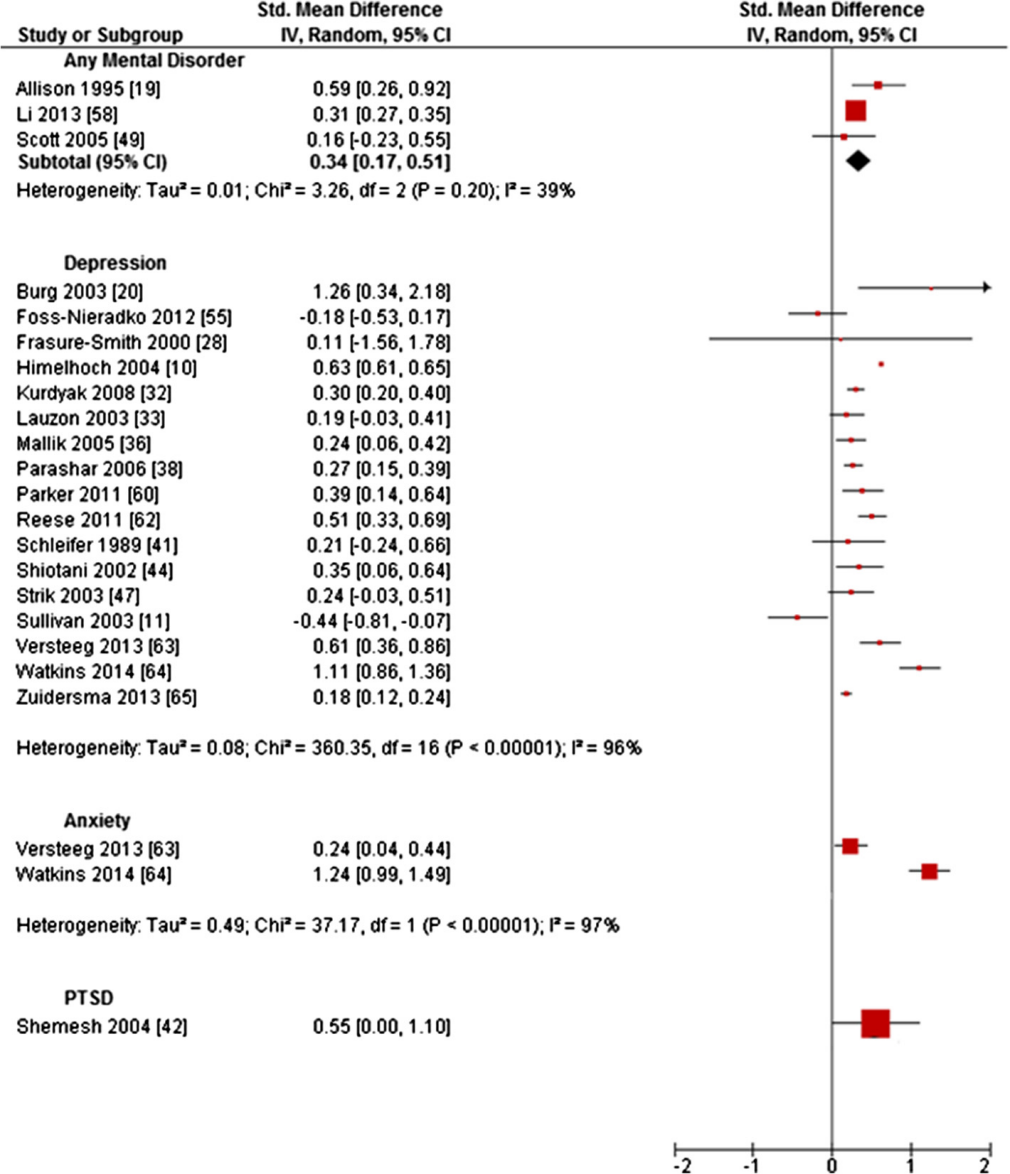
Primary studies regarding *hospital readmission rates.*

**Figure 3:**
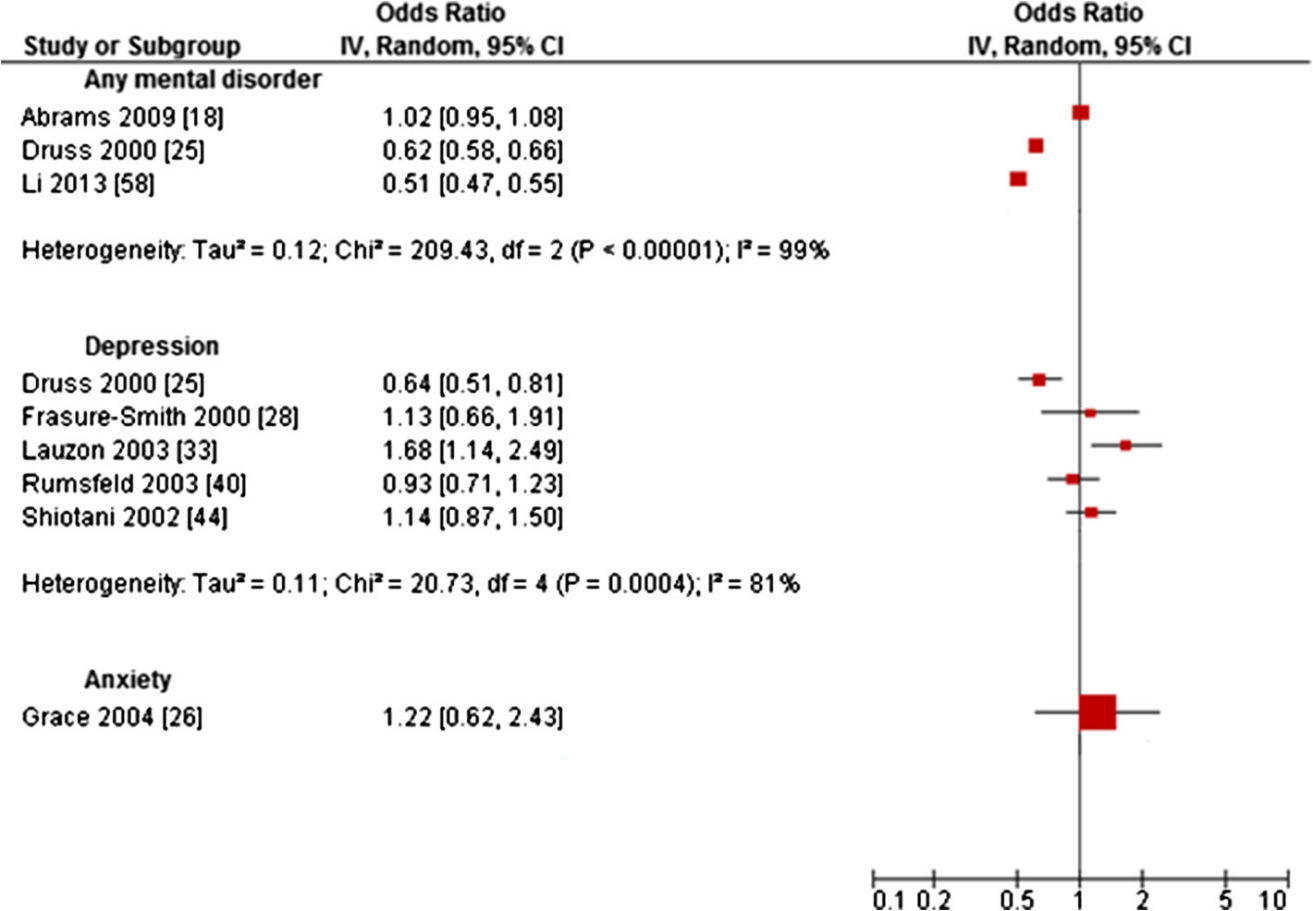
Primary studies regarding *catheterization rates.*

**Figure 4:**
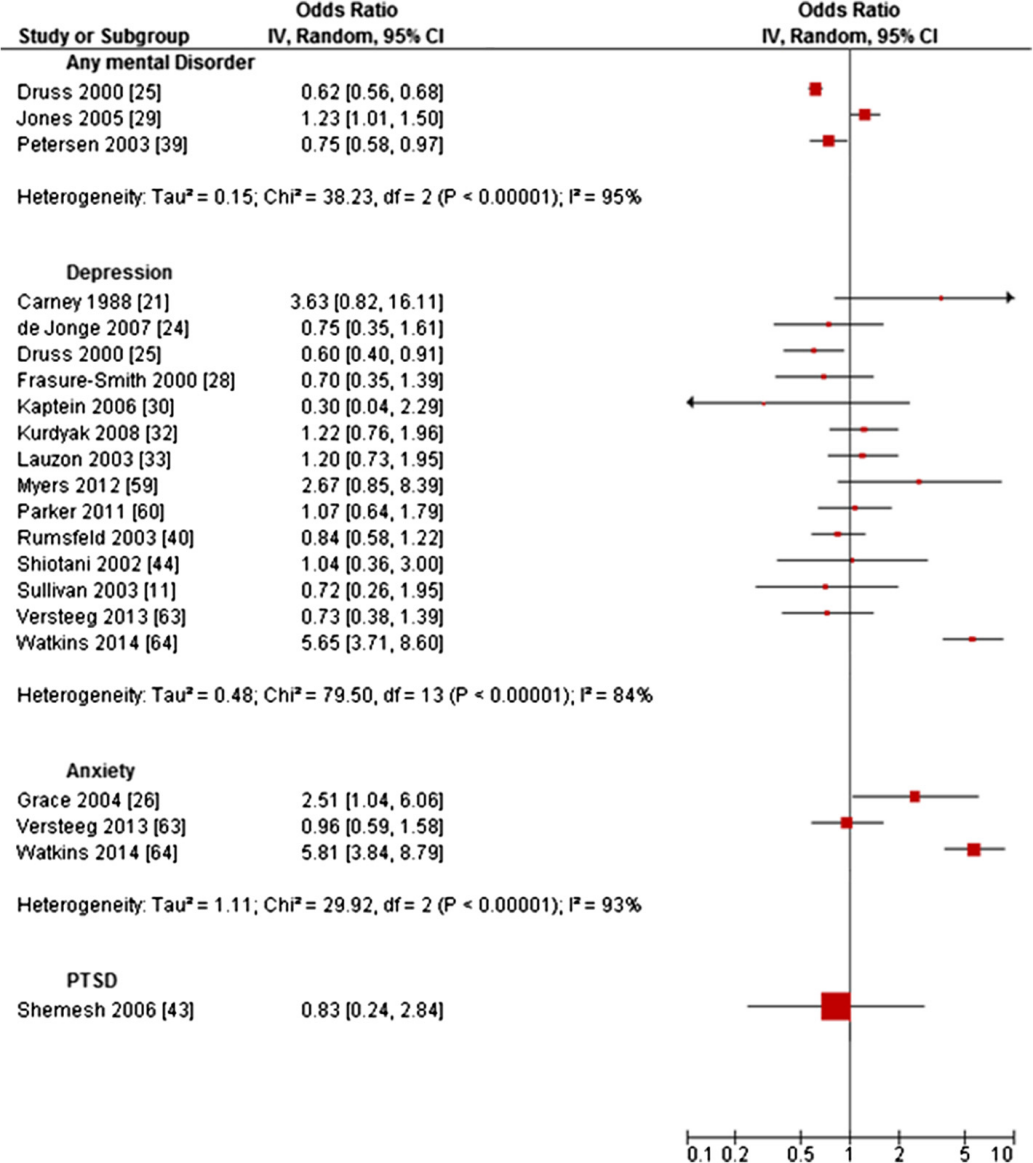
Primary studies regarding rates for *CABG.*

**Figure 5:**
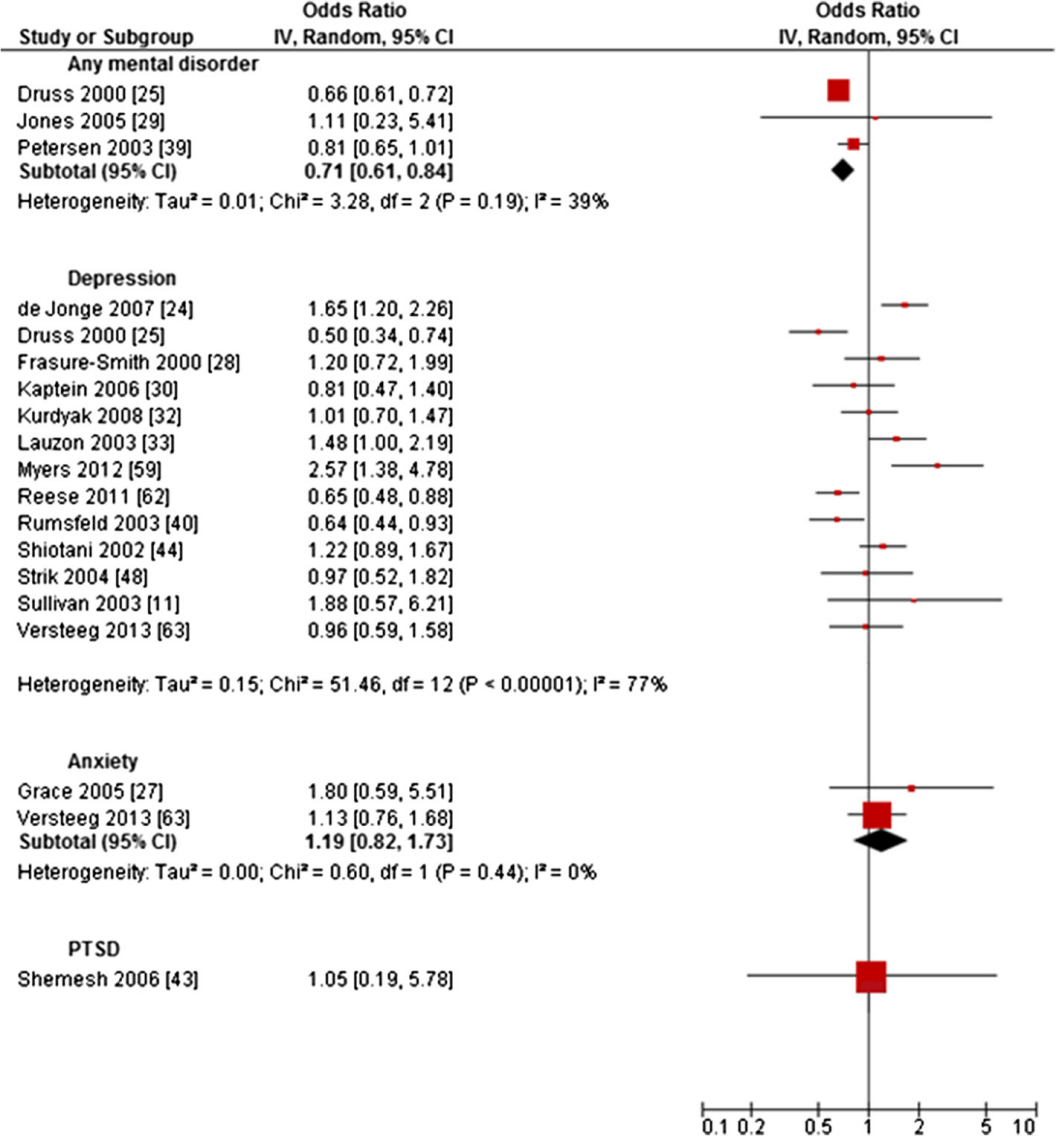
Primary studies regarding rates for *PTCA.*

**Figure 6:**
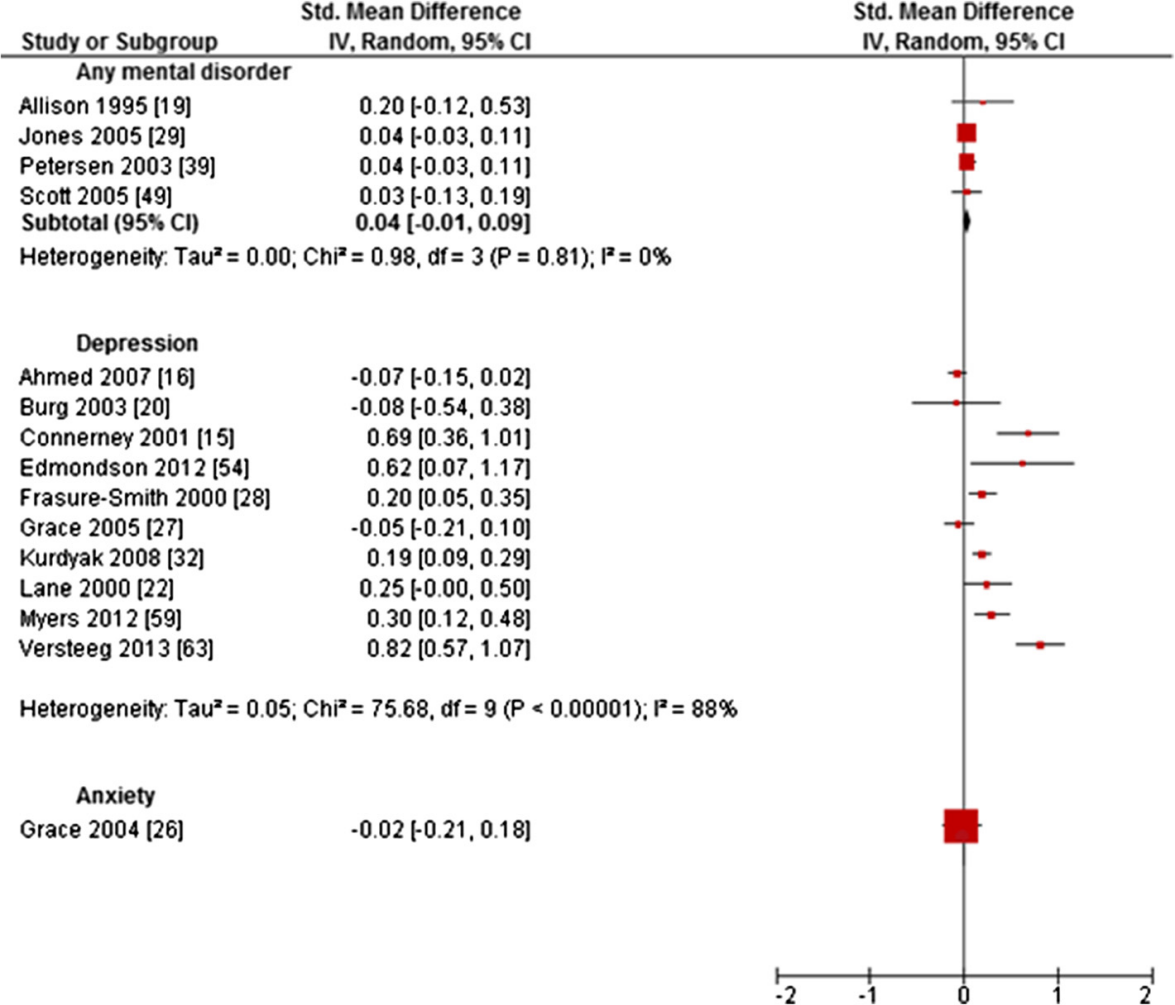
Primary studies regarding *length of index hospital stay.*

Forest plots are reported (Figures 2, 3, 4, 5 and 6) for all outcomes examined in five or more primary studies. Chinn’s method for converting an OR to effect size was used to compute SMDs of continuous outcomes that had been dichotomized in primary studies [12]. Not included in the analysis were studies comparing mentally comorbid patients to patients without mental disorders using beta-coefficients derived from regression analyses, due to their methodological shortcomings when used as measures of effect [13]. Heterogeneity was tested for statistical significance by using Q-statistics (chi-square statistic of heterogeneity test). To examine the extent of heterogeneity, I^2^ was computed. According to the Cochrane Handbook for Systematic Reviews of Interventions, I^2^ of 0%-60% can be regarded as not important to moderate (0–60%), while I^2^ > 60% indicates substantial heterogeneity [14]. Random-effects meta-analyses were conducted for calculating pooled estimates (SMD/OR with 95%-CI), in case of none-substantial heterogeneity.

## Results

The literature search revealed 7275 potentially relevant studies, of which 52 (53 articles) met inclusion criteria (see Table 1) [10,11,15-65].

**Table 1:** **Characteristics of included primary studies**

***#***	***[Ref] First author (Country)***	***Year***	***Study sample***	***Health care costs/Resource utilization***	***Mental disorder (Assessment)***	***n***
1	[18] Abrams (USA)	2009	AMI	Catheterization	Any mental disorder (Database)	21745
2	[16] Ahmed (USA)	2007	CAD	Length of stay	Depression (Database)	2744
3	[19] Allison (USA)	1995	CAD	Readmission costs	Any mental disorder (SCL-90R)	381
				Length of stay		
4	[20] Burg (USA)	2003	CABG	Cardiac readmission rate	Depression (BDI)	89
				Length of stay		
5	[51] Cai (USA)	2013	MI	Readmission	Any mental disorder (Database)	287881
				Length of stay		
6	[21] Carney (USA)	1988	Patients with suspected CAD	CABG rate (at one year postdischarge)	Depression (DIS)	52
7	[15] Connerney (USA)	2001	CABG	Cardiac readmission rate	Depression (BDI)	309
				Length of stay		
8	[23] Dao (USA)	2010	patients undergone CABG	Length of stay	Depression (medical records)	63 061
9	[52] Dao, Youssef (USA)	2010	Patients undergone CABG	Length of stay	Depression (MINI Interview)	358
10	[53] Dao (USA)	2012	Patients undergone CABG	Length of stay	Anxiety (Database)	17885
11	[24] de Jonge (Netherlands)	2007	AMI	PTCA	Depression (BDI,CIDI)	1205
				CABG		
12	[25] Druss (USA)	2000	MI	Catheterization rate (during index admission)	Mood disorders (Database)	113653
				CABG rate (during index admission)	Any mental disorder (Database)	
				PTCA rate (during index admission)		
13	[54] Edmondson (USA)	2012	ACS	Length of stay	Depression (BDI/PHQ-9, DISH)	120
14	[55] Foss-Nieradko (Poland)	2012	Patients undergone CABG	Readmission rates	Depression (BDI)	170
15	[28] Frasure-Smith (Canada)	2000	MI	Readmission rate	Depression (BDI)	848
				Readmission costs		
				Catheterization rate (during index admission and one year postdischarge)		
				CABG rate (during index admission and one year postdischarge)		
				PTCA rate (during index admission and one year postdischarge)		
				Length of stay (Days in intensive care, Days on ward, Days on coronary intensive care, Days on ward for cardiac readmissions)		
				Outpatient visits (Total outpatient visits, Other MD visits, Cardiologist visits Mental health specialist visits)		
				Total emergency department visits, Cost of emergency department visits		
				Total health care costs		
16	[26,27] Grace (Canada)	2004	Unstable Angina/ MI	Catheterization rate (at six month and one year postdischarge)	Anxiety (Anxiety Subscale of the primary Care evaluation of mental disorders, phobic anxiety subscale of the middlesex Hospital Questionaire)	913
				CABG rate (at six month and one year postdischarge)	Depression (BDI)	
				PTCA rate (at six month and one year postdischarge)		
				Length of stay		
				Emergency department visits		
				GP visits		
17	[10] Himelhoch (USA)	2004	CAD	Readmission rate	Depression (Clinical Classification System)	1238895
				Emergency department visits		
18	[29] Jones (USA)	2005	MI	CABG rate (during index admission and 30 days after discharge)	Any mental disorder (Database)	3368
				PTCA rate (during index admission and 30 days after discharge)		
				Length of stay		
19	[30] Kaptein (Netherlands)	2006	MI	CABG rate (during index admission)	Depression (BDI)	475
				PTCA rate (during index admission)		
				Symptoms treated by any healthcare worker		
				Mental health specialist visits		
20	[56] Ketterer (USA)	2010	CAD	CAD-related total costs (year before evaluation)	Any mental disorder (SCL-90R)	164
21	[31] Kronish (USA)	2006	Unstable Angina/ MI	Cardiac rehabilitation attendance	Depression (BDI)	492
22	[32] Kurdyak (Canada)	2008	AMI	PTCA	Depression (BCDRS)	1941
				CABG		
				Length of stay		
				Hospital readmission		
				Family doctor visits		
				Emergency department visits		
23	[57] Kurdyak (Canada)	2011	AMI	PTCA	Depression (BCDRS, short form)	1941
				CABG		
24	[22] Lane (Great Britain)	2000	MI	Length of stay	Depression (BDI)	288
25	[33] Lauzon (USA)	2003	MI	Readmission rate	Depression (BDI)	550
				Catheterization rate (within 30 days and one year postdischarge)		
				CABG rate (within 30 days and one year postdischarge)		
				PTCA rate (within 30 days and one year postdischarge)		
				Length of stay		
26	[34] Levine (USA)	1996	MI	Length of stay	Depression (BDI)	210
27	[58] Li (USA)	2013	AMI	Readmission rate	Any mental disorder (Database)	102783
				Cathetherization		
28	[35] Maeland (Norway)	1989	MI	Readmission rate	Any mental disorder (ADI)	280
				Physician visits		
29	[36] Mallik (USA)	2005	CABG	Readmission rate	Depression (GDS-S)	936
30	[17] McGee (Ireland)	2006	ACS	Physician visits	Depression (HADS-D/BDI)	681
				Cardiac rehabilitation attendance		
31	[59] Myers (Israel)	2012	MI	CABG	Depression (BDI)	632
				PTCA		
				Length of stay		
32	[37] Oxlad (Australia)	2006	CABG	Cardiac readmission rate	Anxiety (DASS)	119
					Depression (DASS)	
					PTSD (PDS)	
33	[38] Parashar (USA)	2006	MI	Readmission rate	Depression (PHQ)	1873
34	[60] Parker (Australia)	2011	ACS	Readmission rate	Depression (CIDI)	489
				CABG		
35	[39] Petersen (USA)	2003	MI	CABG rate (during index admission and in 90 days after discharge)	Any mental disorder (Database)	4340
				PTCA rate (during index admission and in 90 days after discharge)		
				Length of stay		
36	[61] Poole (Great Britain)	2014	Patients undergone CABG	Length of stay	Depression (BDI)	310
37	[62] Reese (USA)	2011	AMI	Readmission rate	Depression (BDI, Depression Interview and Structured Hamilton)	766
				PTCA		
38	[40] Rumsfeld (USA)	2003	Unstable Angina/ MI	Catheterization rate (during index admission)	Depression (Database)	1957
				CABG rate (during index admission)		
				PTCA rate (during index admission)		
39	[41] Schleifer (USA)	1989	MI	Readmission rate	Depression (Schedule for Affective Disorders and Schizophrenia)	283
40	[49] Scott (Australia)	2005	ACS	Readmission rate	Any mental disorder (Database)	2156
				Length of stay		
				Cardiac rehabilitation attendance		
41	[42] Shemesh (Israel)	2004	MI	Cardiac readmission rate	PTSD (IES)	76
42	[43] Shemesh (Israel)	2006	MI	CABG rate (6–9 months after MI)	PTSD (IES)	65
				PTCA rate (6–9 months after MI)		
43	[44] Shiotani (Japan)	2002	MI	Readmission rate	Depression (SDS)	1042
				Catheterization rate (during index admission)		
				CABG rate (during index admission)		
				PTCA rate (during index admission)		
44	[45] Smolderen (USA)	2009	AMI	Readmissions	Depression (PHQ)	481
45	[46] Stern (USA)	1977	MI	Readmission rate	Depression (SDS)	63
46	[47] Strik (Netherlands)	2003	MI	Readmission rate (invasive procedures)	Depression, Anxiety (SCL-90)	318
				Increased health care consumption		
47	[48] Strik (Netherlands)	2004	MI	PTCA rate (during index admission)	Depression (SCL-90)	206
				Increased health care consumption		
48	[11] Sullivan (USA)	2003	CAD	Cardiac readmission rate	Depression (HAM-D/DIS)	198
				CABG rate (after 5 years)		
				PTCA rate (after 5 years)		
				Total health care costs		
49	[50] Tully (Australia)	2008	patients undergoing	Readmissions	Depression	226
			first CABG		Anxiety (DASS)	
50	[63] Versteeg (Netherlands)	2013	CAD	Readmission rate	Depression (HADS-D)	610
				CABG	Anxiety (HADS-A)	
				PTCA		
				Length of stay		
51	[64] Watkins (USA)	2013	CAD	Readmission rate	Depression (HADS-D)	934
				CABG	Anxiety (HADS-A)	
52	[65] Zuidersma	2013	MI	Readmission rate	Depression (BDI, CIDI)	2704

ADI = Anxiety, depression and irritability inventory; BCDRS = Brief Carroll Depression Rating Scale; CIDI = Composite International Diagnostic Interview; DASS = Depression Anxiety Stress Scale; DIS = Diagnostic Interview Schedule for DSM III-R; GDS-S = Geriatric Depression Scale Short Form; IES = Impact of events scale; HADS-D = Hospital Anxiety and Depression Scale – Depression; HAM-D = Hamilton Rating Scale for Depression; PDS = Posttraumatic Diagnostic Scale; PHQ-9 = Patient Health Questionnaire; SCL-90 = Symptom Check List 90; SDS = Zung- Self-Rating Depression Scale.

The majority of studies analyzed depressive disorders and mood disorders (N = 40) [10,11,15-17,20-26,28,30-34,36-38,40,41,44-48,50,52,54,55,57,59-65], followed by *anxiety disorders* (N = 7) [26,37,47,50,53,63,64] and post-traumatic stress disorder (*PTSD)* (N = 3) [37,42,43]. Ten studies investigated *any mental disorder* [18,19,25,29,35,39,49,51,56,58]. Comorbid mental disorders were assessed by *database records* (N = 12) [10,16,18,23,25,29,39,40,49,51,53,58], *clinical interviews* (N = 9) [11,21,24,41,52,54,60,62,65] and *screening questionnaires* (N = 35) [15,17,19,20,22,24,26,28,30-38,42-48,50,54-57,59,61-65]. Health care costs were assessed by *database records* (N = 36) [10,11,15,16,18,20,22,24,29,30,32-34,36,39,40,42-45,47-54,56-59,61-63,65] and *patient self-report* (N = 15) [17,19,21,26,28,31,33,35-38,46,50,60,64]. Two studies did not describe the assessment of health care costs [41,55].

### Inpatient health care

*Hospital readmission rates* were examined in 29 studies (Table 1) [10,11,15,19,20,28,32,33,35-38,41,42,44-47,49-51,55,58-60,62-65] of which 20 reported sufficient data to compute SMDs ranging from −0.44 to 1.26 (Figure 2) [10,11,19,20,28,32,33,36,38,41,42,44,47,49,55,58,60,62-65]. Substantial heterogeneity was showed for depression (I^2^ = 96%) and anxiety (I^2^ = 97%), but not for any mental disorder (I^2^ = 39%). The meta-analysis indicated significantly increased hospital readmission rates for patients with patients with any mental disorder compared to patients without a mental disorder (pooled SMD = 0.34; 95%-CI [0.17;0.51]). Eleven out of 17 primary studies reported significantly increased hospital readmission rates for patients with depression compared to those without (Figure 2). The only study reporting a contrary finding [11], showed no differences between major depression and no depression, while patients with minor depression had the lowest rates of readmission, leading to a significantly lower readmission rate for the combined group of patients with major or minor depression (SMD = −0.44, Figure 2). This U-shaped readmission pattern was not reported by any of the other studies, which mainly did not differentiate between depression severity levels. Both studies on anxiety reported significantly increased readmission rates for patients with increased levels of anxiety compared to those without (SMDs = 0.24 and 1.24, Figure 2). With regard to patients with comorbid PTSD, Shemesh [42] reported significantly increased hospital readmission rates compared to patients without PTSD (SMD = 0.55, Figure 2).

Twenty-two studies collected data regarding *invasive procedures* (Table 1), with eight studies on *coronary catheterization* [18,25,26,28,33,40,44,58], nineteen on *CABG* [11,21,24-26,28-30,32,33,39,40,43,44,57,59,60,63,64] and eighteen on *PTCA* [11,24-26,28-30,32,33,39,40,43,44,48,57,59,62,63].

Results regarding coronary *catheterization rates* proved to be ambiguous with OR ranging from 0.51 to 1.68 [18,25,26,28,33,40,44,58]. Substantial heterogeneity was showed for any mental disorder (I^2^ = 99%) and depression (I^2^ = 81%). On the primary study level, most studies reported non-significant results, with only three studies reporting lower ORs and one study reporting a higher OR for patients with the respective mental disorder compared to those without (Figure 3).

The 18 primary studies investigating *CABG* reported a range of OR from 0.30 to 5.81 (Figure 4) [11,21,24-26,28-30,32,33,39,40,43,44,59,60,63,64]. Heterogeneity was substantial for any mental disorder (I^2^ = 95%), depression (I^2^ = 84%) and anxiety (I^2^ = 93%). On the primary study level, most studies reported non-significant results, with only three studies reporting lower ORs and four studies reporting higher ORs for patients with the respective mental disorder compared to those without (Figure 3).

The 18 primary studies on *PTCA* showed a range of OR from 0.50 to 2.57 (Figure 5) [11,24-30,32,33,39,40,43,44,48,59,62,63]. Heterogeneity was low to moderate for any mental disorder (I^2^ = 39%), low for anxiety (I^2^ = 0%) and substantial for depression (I^2^ = 77%). The meta-analysis showed a significantly decreased odds for *PTCA* in patients with any mental disorder compared to patients without any mental disorder (OR = 0.71 [0.61;0.84]), while the estimate was non-significant with regard to anxiety (Figure 5). Most studies on depression reported non-significant results, while three studies reported lower ORs and three studies higher ORs for patients with depression compared to those without (Figure 5). With regard to patients with PTSD, the result was non-significant too [43].

Twenty-one studies analyzed *length of index hospital stay* [15,16,19,20,22,23,27-29,32-34,39,49,51-54,59,61,63] (Table 1), of which 15 reported sufficient data to compute SMDs ranging from −0.08 to 0.82 (Figure 6) [15,16,19,20,22,26-29,32,39,49,54,59,63]. Heterogeneity was substantial for depression (I^2^ = 88%) but not for any mental disorder (I^2^ = 0%). The meta-analysis showed no significant difference in length of stay between people with and without any mental disorder. Regarding depression, *length of index hospital stay* was significantly increased for patients with depression compared to patients without depression in six primary studies, while four primary studies reported non-significant results. With regard to anxiety, the result was non-significant in one study [26].

### Outpatient health care

*Physician visits* were investigated in five studies (N = 5) [17,26,28,32,35]. Frasure-Smith et al. [28] reported significantly increased total outpatient visits (SMD = 0.27; standard error (SE) = 0.07), significantly increased visits to other medical doctors (SMD = 0.29; SE = 0.07) and significantly increased visits to a cardiologist (SMD = 0.02; SE = 0.07) for CAD patients with depression compared to CAD patients without depression. Kurdyak et al. [32] reported significantly increased cardiologist visits (d = 0.26; SE = 0.05), significantly increased general internist visits (SMD = 0.15; SE = 0.05) and significantly increased visits to a family doctor (SMD = 0.19; SE = 0.05) for CAD patients with depression compared to CAD patients without depression. Grace et al. [26] found no significant differences in general practitioner visits for CAD patients with anxiety compared to CAD patients without anxiety, whereas McGee et al. [17] found an increased rate of visits to a general physician for CAD patients with depression compared to CAD patients without depression (OR = 2.00; 95%-CI: 1.2-3.2).

Studies investigating *mental health specialist visits* (N = 2) [28,30] found significantly more visits for CAD patients with comorbid mental disorders compared to CAD patients without mental disorders (SMD = 0.16: SE = 0.07 [28]; OR = 5.65: 95%-CI: 2.32-13.78 [30]).

With regard to *emergency department visits* (N = 4) [10,26,28,32], Frasure-Smith et al. [28] reported a significant effect size of SMD = 0.23 (SE = 0.07) for *total emergency department visits* and a significant effect size of SMD = 0.25 (SE = 0.07) for *costs of emergency department visits* in CAD patients with depression compared to CAD patients without depression. Himelhoch et al. [10] found significantly increased *emergency department visits* (OR = 2.64; 95%-CI: 2.55-2.73) for CAD patients with depression compared to CAD patients without depression, whereas Grace et al. [26] found no significant differences in *emergency department visits* for CAD patients with anxiety compared to CAD patients without anxiety. Kurdyak et al. [32] found a significant effect size of SMD = 0.20 (SE = 0.05) for CAD patients with depression compared to CAD patients without depression. Between CAD patients with comorbid mental disorders and CAD patients without mental disorders, mainly non-significant differences were found (OR ranged from 0.51 to 0.89) regarding *attendance of cardiac rehabilitation* (N = 3) [17,31,49].

### Total health care costs

*Total health care costs* were examined in five studies [11,28,47,48,56]. Frasure-Smith et al. [28] reported significantly increased *total health care costs* in patients after a myocardial infarction with comorbid depression (SMD = 0.14; SE = 0.07). Strik et al. found in two studies significantly increased *health care consumption* in patients after a myocardial infarction with comorbid anxiety (OR = 2.02; 95%-CI: 1.25-3.25) and depression (OR = 1.61; 95%-CI: 1.00-2.57) [47]; (OR = 1.98; 95%-CI: 1.00-3.93) [48]. Sullivan et al. compared two groups of patients with comorbid mental disorders and reported a U-shaped combination of total five-year median costs of $34,670 for patients with comorbid major depression, $22,183 for patients with comorbid minor depression and $40,193 for patients without comorbid depression [11]. Ketterer et al. [56] found significantly increased hospital costs for CAD patients with anxiety (normal anxiety level: $8,505; moderately elevated $8,736 and significantly elevated: $12,022) and phobic anxiety ($8,646; moderately elevated $8,276 and significantly elevated: $13,052) but not for increased levels of somatization, obsessive-compulsiveness or depression [56].

## Discussion

The present meta-analysis is perhaps the first to comprehensively summarize the impact of comorbid mental disorders in CAD patients on a broad range of direct cost parameters amongst inpatients and outpatients. The results generally indicated a negative impact of comorbid mental disorders on hospital readmission rates. Although there were fewer studies reporting outpatient costs such as physician visits, mental health specialist visits, emergency department visits and attendance of cardiac rehabilitation programs, meta-analyses indicated increased costs in CAD patients with comorbid mental disorders.

The finding relating to increased hospital readmission rates is generally consistent with previous reviews of a negative impact of depression on cardiovascular events and mortality in myocardial infarction [66,67]. The higher readmission rates in CAD patients with comorbid mental disorders may at least partly reflect the increased proportion of comorbidities, severity of atherosclerosis, and perhaps the delay in seeking medical care in patients with mental disorders [68]. The severity of stenosis in the left main coronary artery, extent of diffuse coronary disease unable to be revascularised, proportion of non-patent grafts, and number of somatic comorbidities have been shown to be associated with an increased risk of suffering from comorbid mental disorders [11,66,67]. This raises the question of whether the higher readmission rates are better explained by disease complications and somatic comorbidities than by comorbid mental disorders, a critique described by Nicholson et al. [69] with respect to left ventricular function. However, primary studies of the present review also reported a significant association between mental disorders and health care costs after adjusting for somatic comorbidities or disease severity [10,15,20,25,28,29,31-33,36,38,45,50], indicating that mental comorbidity is an independent predictor of health care costs in CAD.

Nonetheless, CAD patients with documented severe mental comorbidity require dedicated mental health care, potentially leading to higher primary health care costs (e.g. for medication review, metabolic monitoring, specialist referral) but also emergency department visits (e.g. suicidality). Although, based on the finding indicating significantly higher hospital readmission rates, one might also expect higher rates of cardiac-related diagnostic and revascularization procedures, however, findings were inconsistent. A potential explanation relates to evidence indicating that persons with mental disorders are suboptimally treated or referred for diagnostic procedures, less likely to receive optimal coronary revascularization strategies and are susceptible to patient-physician communication barriers [70,71]. Another possible explanation would be, that differences in invasive procedures may vary between patients samples (e.g. acute MI vs. CAD sample). However, there were no clear pattern derivable from comparing the characteristics (Table 1) of those studies which reported significantly lower invasive procedure rates for people with mental disorders compared to those which reported significantly higher procedure rates for this group. Thus, it seems more likely, that invasive procedure rates do not vary substantially between CAD patients with and without mental disorders.

Notwithstanding the increased readmission rate among persons with depression or any comorbid mental disorder, it was found that length of index hospital stay in CAD patients with comorbid mental disorders was generally not increased compared to CAD patients without mental disorders, and only marginally increased in most studies on depression. Although multiple factors likely influence length of index stay, marginally increased length of stay among depressed persons is perhaps a broad marker for their propensity toward postoperative complications, especially post-revascularization stroke, renal failure and deep sternal wound infection [72]. Other potential explanations may relate to accommodation factors such as the requisite need for supported care, awaiting accommodation in rehabilitation facilities, and transfer to rural hospital centres. These well established factors which one would expect to be more strongly associated with an increased length of stay in CAD patients with mental disorder, might be leveled out by the use of Diagnosis Related Groups (DRGs), which limit the length of stay according to the primary diagnosis at admission. Indeed, CAD patients with comorbid mental disorders might need a longer period of convalescence to recover physically to the same degree as CAD patients without mental disorders, and potentially have their mental illness exacerbated during a period of hospitalization or after the rigors of invasive procedures. Consequently, this might increase the probability of using healthcare services after discharge for CAD patients with comorbid mental disorders and might explain both the higher readmission rates and the higher outpatient and overall costs. Another possibility which we were not able to evaluate is that premorbid and postmorbid onset mental disorder may directly influence resource use differently. For example, some evidence suggests that postmorbid onset depression is associated with significantly higher morbidity than premorbid onset depression [60]. Also, recently we observed that years since panic disorder onset was associated with longer length of cardiovascular admissions stay [73].

Primary studies mainly investigated depression. It cannot be assumed that findings relating to depression and CAD costs are generalizable across depression subtypes [74,75]. Moreover, the results of this review cannot be generalized to other mental disorders, as relationships to health care costs might differ depending on the mental disorder under study [5]. For example, worries about one’s health as a symptom of anxiety disorders can lead to both an adequate utilization of our health care services in case of justified worries and an over-utilization of health care services in case of unsubstantiated extensive worries. Similarly, avoidance behavior as a characteristic of anxiety might be associated with both over- and under-utilization of health care services depending on the presence of health issues in need of treatment [5,76]. Future research should clarify the relationship of comorbid mental disorders other than depression with health care costs in CAD patients, including more severe mental disorders such as personality disorders, substance abuse, psychoses, and bi-polar disorders.

With regards to the methodological aspects of the original investigations each of the studies utilized varying levels of methodological rigor, and consequently may have introduced heterogeneity in cost estimates. For example, some studies used patient-reported data on health care utilization, which despite evidence of moderate to high reliability of subjective reports [77,78], may not be reliable in persons with mental disorders. Previously we highlighted that depressed patients with somatic diseases tend to misclassify their disease status [1] and certainly, the caveats of self-reported disease status among persons with anxiety disorders is notably prone to bias [79,80]. Such biases in self-reported health care utilization data would consequently lead to heterogeneity in our comparisons between patients with and without mental disorder. Other methodological limitations of the original studies relate to the assessment of comorbid mental disorders predominantly based on screening questionnaires which would potentially comprise patients with subthreshold syndromes. Previous studies investigating depression along a continuum from no depression, minor to major depression showed a U-shaped association with cost parameters [36,41] which may not have been evident in our analyses dichotomizing patients with various severity thresholds. Other possible sources of methodological heterogeneity may include that primary studies stemmed from several different countries with different public health care systems. It has been shown that costs due to hospital admissions vary between different countries [66]. Even within the same country, it is possible that the setting (inpatient or outpatient) has an impact on prevalence rates of mental disorders and may thus also impact the association with health care costs in CAD patients [18,81].

Some methodological limitations should be taken into account, when reading the results of this systematic review. First, considering that we restricted the search for eligible studies in the English and German language the selection process of primary studies may have been biased towards studies in only two languages. Publication bias may have occurred and it remains unclear to what extent non-significant results were not published in the first instance or retrieved by our search strategy (e.g. doctoral theses). Furthermore, from a database search yielding 7273 articles the preliminary selection was undertaken by one reviewer only, although our secondary screening process involved two reviewers. Second, the analytical synthesis of the health care cost data was hampered by methodological and statistical heterogeneity of the included primary studies. The primary studies differed regarding the selection of the sample (e.g. clinical or population-based samples), the assessment of comorbid mental disorders (standardized interview, screening questionnaire, self-report), the comparability of the groups (adjustment of relevant confounding variables), and the assessment of cost outcomes (monetarily/resource utilization). To avoid leveling out varying costs across studies, we only conducted meta-analyses in case of none-substantial statistical heterogeneity. Moreover, some studies did not provide sufficient data to compute effect sizes and it remains an open question whether including the results of these studies would have changed the finding of the present review substantially. Notwithstanding these limitations, this review constitutes a comprehensive and representative view on direct costs attributable to comorbid CAD and mental disorders.

## Conclusion

In conclusion these data suggest that comorbid mental disorders in CAD patients are associated with an increased healthcare utilization and costs consistent with previous meta-analyses on other somatic diseases [4-6]. Together with increased work loss days in CAD patients with mental disorders [82], the present study highlights the public health relevance of comorbid mental disorders in CAD patients. Even if increases in health care costs may be justified in some cases and not always indicative for over-utilization, the present finding point to the need of optimizing health care for people with CAD and mental disorders by improving the diagnosis and treatment of comorbid mental disorders in patients with CAD [83-85]. Recommendations for clinical practice are to recognize mental disorders in an early stage of treatment, redistribute the allocation of combined mental and primary health care services for comorbid CAD and mental disorder, and to ultimately minimize incremental costs. Thereby, comprehensive approaches such as blended collaborative care interventions [86-88] might help to bridge the gap between medical and psychosocial interventions and thus improve the effectiveness of health care in CAD patients. The limited number of cost-effectiveness studies on treating mental disorders (only examined for depression) in CAD patients conducted so far indicate that societal costs are at least not increased by adding an evidence-based psychotherapy, pharmacotherapy or collaborative care intervention to standard CAD health care [85,86]. Based on preliminary results of an ongoing systematic review it further seems as collaborative care interventions for people with CAD and depression might not only significantly reduce depressive symptoms, but also major cardiac events [89]. If this proves to be true, treating depression might help to lower the increased readmission rate for people with CAD and depression reported in the present study and thus reduce health care expenditures in the long term.
